# Protective effect of *Asarum sieboldii* essential oil on ovalbumin induced allergic rhinitis in rat

**DOI:** 10.1042/BSR20191370

**Published:** 2020-06-02

**Authors:** Zhicheng Zhang, Haoran Kang

**Affiliations:** Department of Otorhinolaryngology, The First Affiliated Hospital of Henan University of Chinese Medicine, Zhengzhou 450000, People’s Republic of China

**Keywords:** cytokines, histamine, Nasal mucosa, nasal scratching, sneezing

## Abstract

**Background:** The study was aimed to investigate the protective effect of *Asarum sieboldii* Miq. essential oil (AEO) on ovalbumin (OVA)-induced allergic rhinitis (AR) in rats.

**Methods and results:** Sixty Sprague–Dawley male rats were randomly divided into six groups (*n*=10): control, model, cetirizine (Cet, 4.65 g/kg), and AEO (0.5, 1.5, 3 g/kg) groups. All animals except the control group received repeated intranasal instillation with 20 μl of 20% OVA in Al(OH)_3_ saline solvent for 15 days. The control group was intranasally instilled with 5 mg/ml of Al(OH)_3_ instead of the same procedure. In the 15 days, Cet and AEO were orally administrated for 28 days. At the end of the drug administration, 20 μl of 5% OVA was given to animals to stimulate allergic reaction, then the rat behavioral detection, assessment of the patho-morphological changes in nasal mucosa, and the serum biomarkers were determined. The result showed that AEO could significantly reduce the amount of nasal secretions, sneezing, and the degree of nasal scratching in AR rats with EC_50_ = 1.5 and 2.8 g/kg, respectively. The degree of nasal mucosal inflammation in AEO group improved, the levels of immunoglobulin E (IgE), histamine, IL-4, IL-5, IL-17 were decreased, and the level of IFN-γ was increased obviously with EC_50_ = 2 g/kg.

**Conclusion:** The study suggested that the possible mechanism might be related with the inhibition of histamine release and regulation of the cytokine levels, which plays an important role in the treatment of AR.

## Introduction

Allergic rhinitis (AR) is a symptomatic disorder of the nose, which is induced after allergen exposure by an immunoglobulin E (IgE)-mediated inflammation of the membranes [1]. The prevalence of self-reported AR has been estimated to range from 2 to 25% in children and from 1 to 40% in adults. The prevalence of confirmed AR in adults in Europe ranges from 17 to 28.5% [2]. Recent studies have shown that the prevalence of AR has increased from 11.1 to 17.6% in Chinese adults [3]. AR is also associated with comorbidities such as asthma, rhinosinusitis, conjunctivitis, nasal polyposis, and otitis media; AR has been confirmed to be a risk factor for asthma in both adults and children [1]. Indeed, a recent study has confirmed that AR is also a risk factor for both acute and chronic rhinosinusitis [4]. This may be of particular relevance in pediatric patients, as the pivotal pathophysiologic sites are the ostiomeatal complex, the sphenoethmoidal recess and the Eustachian tube, which are linked with otitis media and other sinus-related conditions in infants and children [5,6].

Traditionally, AR is diagnosed with the combined evaluation of history, allergen diagnosis such as *in vivo* skin prick tests (SPTs) and *in vitro* allergen-specific IgE in serum to confirm the correlation between the characteristic symptoms, such as sneezing, nasal itch, rhinorrhea and nasal blockage, and potential allergens [7–9]. Until now, the AR has not been used as complete therapy, the goal of the therapy is just relief from the symptoms. Therapeutic options available to achieve this goal include avoidance measures, nasal saline irrigation, oral antihistamines, intranasal corticosteroids, combination intranasal corticosteroid/antihistamine sprays, leukotriene receptor antagonists (LTRAs), and allergen immunotherapy [10–13]. Thus, finding an effective drug for AR is an urgent and important thing for the patients.

Recent research shows that plant essential oils may be an alternative therapy method for AR, and many of them are much more effective and safer than chemical reagents. *Asarum sieboldii* Miquel (Aristolochiaceae), with a Chinese name Xixin (XX), is a perennial plant that is widely distributed in China, Korea, and Japan [14,15]. It is a traditional medicine in China, which has been used for a long time. Previous phytochemical investigation on XX resulted in the identification of compositions of the volatile oil [16], phenolics, lignans, and amide [17]. Current studies have shown that the essential oil of XX has many pharmacological properties, such as anti-inflammatory, antibacterial, and anti-allergic, as well as affecting the respiratory and circulatory systems [18]. However, few studies have examined the potential effect of the essential oil of XX on AR, thus, in the present study, we evaluated the protective effect of the essential oil of XX on ovalbumin (OVA)-induced AR in rats, in order to find a potential therapy method for AR.

## Materials and methods

### Reagents

OVA was obtained from Sigma (batch number: 094K1224); cetrizine (Cet) tablets were purchased from Chengdu Heng Rui Pharmaceutical Co., Ltd (China, batch number: 20170202). Rat IgE ELISA kit (Cloud-Clone Corp., Wuhan, China, batch number: SEA545Ra). Histamine ELISA kit (Cloud-Clone Corp., Wuhan, China, batch number: CEA927Ge); IL-4 ELISA kit (Cloud-Clone Corp., Wuhan, China, batch number: SEA077Ra); IL-5 ELISA kit (Cloud-Clone Corp., Wuhan, China, batch number: SEA078Ra); IL-17 ELISA kit (Cloud-Clone Corp., Wuhan, China, batch number: SEA063Ra); IFN-γ ELISA (Cloud-Clone Corp., Wuhan, China, batch number: bSEA033Ra); Hematoxylin–Eosin (H&E) staining solution (Nanjing Jiancheng Bioengineering Research Institute, Nanjing, China, batch number: i032).

### Preparation of *Asarum sieboldii* Miq. essential oil

The root of *Asarum sieboldii* was purchased from Guangdong Health Care Chain Pharmacy Co., Ltd. (Guangdong, China) on 10 January 2017, with the batch number 20170110. The plant was verified by Professor Weisheng Feng (College of Pharmacy, Henan University of Chinese Medicine, Henan, China). The samples were washed three times with distilled water and dried in an oven at 4°C for 3 days and then pulverized into fine powder. The extract of *Asarum sieboldii* Miq. essential oil (AEO) was prepared according to Chinese Pharmacopoeia (Appendix XD ‘Volatile Oil Determination Method’ in 2015 edition). The powder of the crude drug was extracted with petroleum ether twice by Soxhlet extraction, and then filtered. The combined filtrate was dried using anhydrous Na_2_SO_4_ and concentrated under vacuum at 50°C using a rotary evaporator. The oil yield was 2.2%, then the extracted oil was calculated and diluted with Tween-80 and water (Oil:Tween-80:water = 1:1:8) and obtained 0.2 g crude drug/ml, then stored at 4°C for use.

### Animals

Sixty male Sprague–Dawley (SD) rats (body weight: 200–220 g) were obtained from the Experimental Animal Center of Henan University of Chinese Medicine. Six rats were kept in single polyacrylic cage and were quarantined for 1 week before the experiments. The whole experiment was carried out at the Experimental Animal Center of Henan University of Chinese Medicine. All animals were housed under standard controlled conditions (temperature: 24 ± 1°C, humidity: 50 ± 5%, and 12-h light/dark cycle), with free access to food and water, and received human care according to National Institutes of Health Guidelines of the United States (National Research Council of United States, 1996) and the related ethical regulations of Henan University of Chinese Medicine (number: ZZ-201801).

### Experimental design

Sixty male rats were randomly divided into six groups (*n*=10): control group, model group, Cet tablets group (4.65 g/kg), and the AEO extracts groups (0.5, 1.5, 3 g/kg). All the animals except the control group received repeated intranasal instillation of 20% OVA in Al(OH)_3_ saline solvent with 20 μl for 15 consecutive days. The control group were intranasally instilled with 5 mg/ml Al(OH)_3_ instead of the same procedure. After the 15 days, Cet and three dosages of AEO were administrated with the indicated dose through oral administration, once a day for 28 days. The negative control group and the model group were given the same amount of double-steamed water, once a day for 28 days. At the end of the drug administration, 20 μl of 5% OVA was given to the animals except control group to stimulate the allergic reaction, and then the behavior of the rats was detected. The animals were anesthetized with isoflurane gas through anesthesia machine. The blood and serum were obtained from fundus venous plexus for biomarker detection. At the end of the experiment, the animals were killed through cervical dislocation, the nasal mucosa was observed for histo-pathomorphological evaluation.

### Behavioral assessment

After the last 5% OVA stimulation, each animal was observed for 30 min and scored according to the numbers of scratching nose, the number of sneezes, and the degree of runny nose [19–21]. Scratching nose: gentle nose flinch, 1 point; frequent nose flinch, 2 points; double front arms constantly scratching nose, 3 points. Sneeze: 1–3 sneezes, 1 point; 4–10 sneezes, 2 points; 11 or more than 11 sneezes, 3 points. Runny nose: flow to the anterior nostrils, over the front nostrils and full of runny face were scored as 1, 2, and 3 points, respectively. The above three symptom scores were superimposed, and the total score more than 5 points regarded as the model established successfully.

### Histopathological analysis

Nasal mucosa specimens were fixed in 40 g/l formaldehyde solution overnight. The fixed specimens were embedded in paraffin, cut into 5-μm-thick sections and then stained with H&E in terms of the routine histopathological examination. The final stained sections were photographed under a light microscope (BX-50 Olympus) at 200× magnification.

### The levels of biomarker detection

The levels of IgE, histamine, IL-4, IL-5, IL-17, and IFN-γ were detected by ELISA kit, according to the protocol of the related kit, which was obtained from the company, Cloud-Clone Corp, Wuhan, China.

### Statistical analysis

Data were expressed as mean ± SD and all statistical comparisons were made by means of one-way ANOVA test followed by Dunett’s *t* test. *P*<0.05 and <0.01 were considered statistically significant. All calculations were performed using SPSS 19.0 (IBM Corp., Armonk, NY, U.S.A.).

## Results

### General information

**Figure 1:**
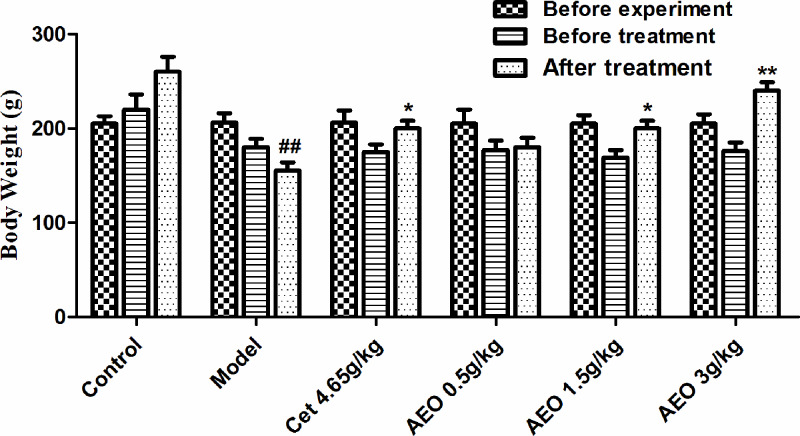
Body weight changes in the process of the experiment of all the rat groups (*n*=10) ^##^*P*<0.01 *vs* control group; *P<0.05, **P<0.01 *vs* model group.

The diet and daily behavior of the animals in control group were basically normal, the hair was bright as usual, and there were no obvious symptoms such as sneezing, nose scratching, and runny nose. In the model group, one rat died. Before death, we observed that the conjunctiva of both eyelids was congested, and the activity of the rat was poor, which was considered as the allergic reaction that led to death. Another three rats developed transient laryngeal wheezing. The other rats in the model group were all less active, had wet anterior nostril and wet eyelid conjunctiva, which was often accompanied by sneezing and nose scratching. And some rats even had bloody nose in the nostrils after stimulation by OVA. In addition, the body weight of the rats in the model group was significantly reduced, compared with the control group (Figure 1). While the rats in the treatment of Cet tablets and AEO groups showed more activity and less symptoms of sneezing, nose scratching, and runny nose. The body weight of the rats in the drug-treated groups was also increased compared with model group (Figure 1).

### Behavioral assessment

**Figure 2:**
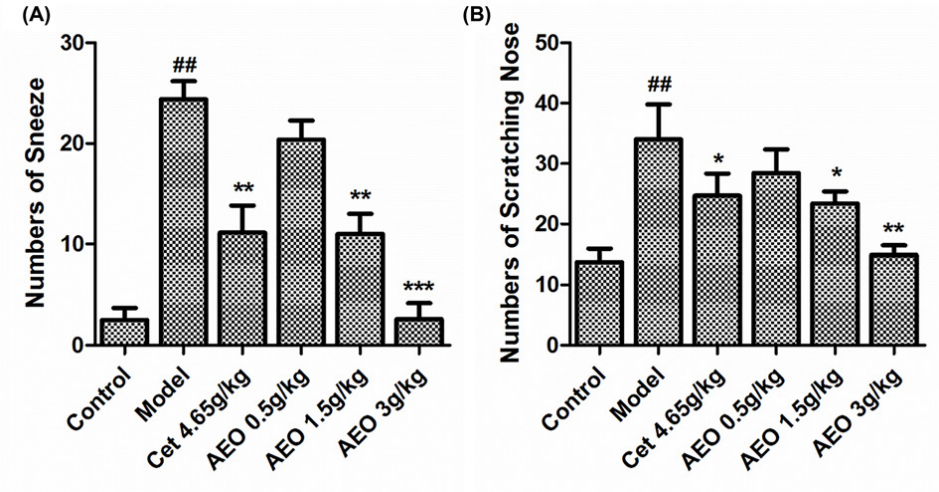
Statistical analysis for the numbers of sneeze and scratching nose in all the rat groups (*n*=10) ^##^*P*<0.01 *vs* control group; *P<0.05, **P<0.01, ***P<0.001 *vs* model group.

As Figure 2 shows, after the last stimulation, there were significant differences in the number of sneezes and nose scratches between model and control groups (Figure 2A,B, *P*<0.01). While the number of sneezing and nose scratching in the Cet (4.65 g/kg) group, AEO high (3 g/kg) and middle (1.5 g/kg) dose groups were significantly lower than that in the model group (*P*<0.05, *P*<0.01, *P*<0.001). The AEO low (0.5 g/kg) dose group showed no significant difference, compared with the model group.

### Morphology changes of the structure of nasal mucosa in rats

**Figure 3:**
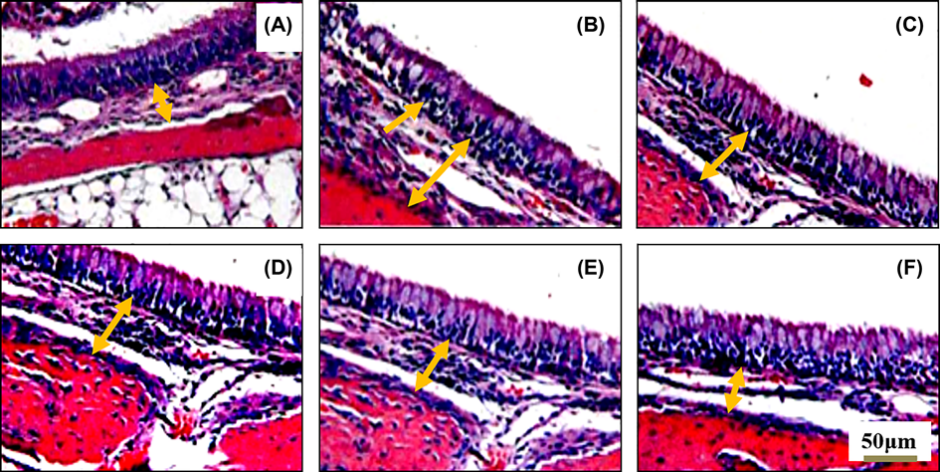
Morphology changes of the structure of nasal mucosa in rats of each group (H&E staining, 200×, scale bar 50 μm) (**A**) Control group, (**B**) Model group, (**C**) Cet tablets group (4.65 g/kg), (**D**) AEO extracts groups (0.5 g/kg), (**E**) AEO extracts groups (1.5 g/kg), (**F**) AEO extracts groups (3 g/kg). Single arrowhead indicated the disordered epithelial cells; double arrowhead indicated the dense fibrous connective tissue of the submucosa.

In Figure 3, the results showed that the structure of the nasal mucosa in the control group was intact and smooth, the dense fibrous connective tissue of the submucosa is thinner and the gland size is normal (Figure 3A). The structure of nasal mucosa in model control group was disordered, the cilia were different in thickness, the epithelial cells were arranged disorderly, goblet cells were increased, the glands and blood vessels around the submucosa were dilated and proliferated, there were congestion and tissue edema, and inflammatory cells such as eosinophils infiltrated, showing the characteristics of mucosal remodeling (Figure 3B). The mucosal edema and eosinophilic granulocyte decreased in the Cet positive group, AEO medium dose and AEO high dose group (Figure 3C,E,F). The edema and eosinophil infiltration in AEO low-dose group (Figure 3D) had no significant difference compared with the model group.

### Effects of AEO on serum levels of IgE and histamine

As Table 1 shows, the serum IgE in the model group was significantly higher than that of the control group (*P*<0.01). The serum IgE in the AEO high, middle dose group, and Cet group were significantly lower than that of the model group (Table 1, *P*<0.05, *P*<0.01). The content of histamine in control group was 18.63 ng/ml, and that in model group was 112.89 ng/ml, which was significantly higher than that in control group (Table 1, *P*<0.01). The levels of serum histamine in AEO high, middle, low dose groups and Cet group were lower than those in model group, and there were significant differences between AEO high dose group and Cet group, compared with model group (Table 1, *P*<0.05, *P*<0.01).

**Table 1:** Effect of AEO on serum levels of IgE and histamine

Group	IgE (ng/ml)	Histamine (ng/ml)
Control	22.02 ± 3.20	18.63 ± 4.02
Model	110.23 ± 23.51^*^	112.89 ± 36.19^*^
Cet 4.65 g/kg	91.23 ± 16.29	57.13 ± 10.44*
AEO 0.5 g/kg	81.52 ± 15.36	82.27 ± 10.55
AEO 1.5 g/kg	73.23 ± 10.21^†^	67.52 ± 6.16^†^
AEO 3 g/kg	56.86 ± 7.15^‡^	40.11 ± 6.09^‡^

* *P*<0.01 *vs* control group.

† *P*<0.05.

‡ *P*<0.01 *vs* model group.

Data are expressed as the mean ± SD (*n*=10) in each group.

### Effects of AEO on serum levels of IL-4, IL-5, IL-17, and IFN-γ

In Table 2, the levels of serum IL-4 in model group were higher than in control group (*P*<0.01). The levels of serum IL-4 in AEO high, middle, low dose groups and Cet group were significantly lower than in model group (Table 2, *P*<0.05, *P*<0.01). The level of serum IL-5 in model group was higher than that in control group (*P*<0.001). The level of serum IL-5 in AEO high dose group was significantly decreased (Table 2, *P*<0.01). The level of serum IL-17 in the model group was significantly higher than that in the control group (Table 2, *P*<0.001). The levels of serum IL-17 in different doses of AEO and Cet groups were decreased (Table 2, *P*<0.01, *P*<0.001). The levels of serum IFN-γ in model group were lower than those in control group (Table 2, *P*<0.01). The levels of serum IFN-γ in AEO high, middle, low dose group and Cet group were significantly increased in different degrees, compared with the model group (Table 2, *P*<0.05, *P*<0.01, *P*<0.001).

**Table 2:** Effect of AEO on serum levels of IL-4, IL-5, IL-17, and IFN-γ

Group	IL-4 (pg/ml)	IL-5 (ng/ml)	IL-17 (pg/ml)	IFN-γ (pg/ml)
Control	148.85 ± 20.30	1.13 ± 0.02	104.69 ± 5.11	154.69 ± 10.11
Model	355.48 ± 39.51^*^	5.22 ± 0.69^†^	3404.28 ± 77.42^†^^,^^‡^	63.28 ± 5.42^‡^
Cet 4.65 g/kg	154.93 ± 10.29^§^	4.13 ± 1.14	1883.56 ± 110.14^§^	107.16 ± 9.14^§^
AEO 0.5 g/kg	202.22 ± 19.36^‡^	4.97 ± 0.55	1993.90 ± 30.23^§^	101.98 ± 6.23^‡^
AEO 1.5 g/kg	174.33 ± 22.21^‡^	4.02 ± 0.66	1263.35 ± 100.11^§^	104.75 ± 3.11^§^
AEO 3 g/kg	111.26 ± 10.15^§^	3.31 ± 0.09^§^	929.98 ± 39.20^║^	145.18 ± 2.20^║^

* *P*<0.01 *vs* control group.

† *P*<0.001 *vs* control group.

‡ *P*<0.05.

§ *P*<0.01.

║ *P*<0.001 *vs* model group.

Data are expressed as the mean ± SD (*n*=10) in each group.

## Discussion

The typical clinical symptoms of AR are sneezing, nasal mucus, nasal itching etc [22]. Nasal behavior in animal models is an intuitive indicator of pharmacodynamic evaluation. In the present study, the efficacy of AEO was evaluated preliminarily, according to the nasal behavior of rats. The results showed that after 28 days of intragastric administration, AEO significantly reduced the frequency of sneezing and nose scratching, especially in AEO high dose group, while the effect of the low dose group was close to the ineffective dose. Compared with the positive group, the Cet group also had a significant effect after 28 days of administration, which was consistent with the previous report. The results suggest that AEO can effectively improve the nasal allergic symptoms in rats.

Eosinophils and mast cells are the main effector cells of AR. There are high-affinity IgE-binding sites on the surface of mast cells, and a large number of pro-inflammatory factors and vasoactive mediators are stored in the cytoplasm [23]. Eosinophilic granulocytes in peripheral blood and nasal secretions are increased by the release of mast cell degranulation mediators. Eosinophils can damage the epithelium and cilia of the respiratory tract, free histamine and other mediators in mast cells, thereby promoting the development of allergy. The present study found that AEO can reduce the number of mast cells and eosinophils in nasal mucosa, alleviate the infiltration of eosinophils in nasal dry membrane, and control the development of AR.

IgE is the main antibody causing transient hypersensitivity. The body is stimulated by allergen and produces IgE antibody. Therefore, serum IgE level is a marker of allergic diseases such as AR and allergic asthma [24]. IgE can bind to the high-affinity IgE receptor on the surface of basophils and mast cells to sensitize them, and induce the release of inflammatory mediators such as histamine and leukotriene when exposed to the same allergen again. IgE-mediated mast cell activation is one of the mechanisms of transient hypersensitivity. Therefore, serum IgE is one of the important indicators to evaluate the efficacy of anti allergic drugs and explore the mechanism of action. These results suggest that AEO can effectively reduce the IgE content in serum and nasal lavage fluid of rats. At present, traditional Chinese medicine has prominent advantages in this respect.

Histamine is released by the contact of mast cells and basophils with allergens, and is the most important mediator in the nasal allergy. Histamine activates the H1 receptor on vascular endothelial cells of respiratory smooth muscle cells, which induces the increase in C-GMP in the cells, thus producing pro-inflammatory effects, resulting in small vessel dilatation, microvascular permeability, and increased gland secretion [25,26]. These are the important material basis of nasal mucosal edema, increased nasal respiratory resistance, and increased secretion in patients with AR. Oral antihistamines are also recommended as a first-line treatment for AR. These results suggest that AEO has an antihistamine effect, and the improvement of nasal symptoms in AR model rats may be directly related to its antihistamine effect.

The research on the pathogenesis of AR in modern medicine is mainly the classic Thl/Th2 balance theory. IFN-γ, IL-4, and IL-5 are Th1 and Th2 cell-related cytokines, respectively, IL-17 is Th17 cell-related cytokines [27]. When specific antigens act on specific individuals, antigen-presenting cells transmit antigenic signals to T cells to offset Th cell differentiation, i.e. Thl reaction is biased toward Th2 reaction, and IL-4 and IL-5 secretion is increased. IL-4, in turn, promotes mast cell differentiation and maturation, cooperates with IL-13 to promote B-cell transformation into plasma cell synthesis and build immune memory, and rapidly synthesizes more allergen-specific IgE when allergens are re-stimulated. Therefore, IL-4, an important cytokine up-regulating IgE, IFN-γ, is considered to be an anti-allergic factor of respiratory tract and a biological antagonist of IL-4 synthesis. IL-5 plays a key role in stimulating eosinophil activation and maturation, prolonging survival time, and is an important factor in delayed phase hypersensitivity [28]. Studies have shown that IL-5 mediated eosinophil infiltration in the trachea is a prerequisite for airway hyperresponsiveness [29]. The results of this study showed that IL-4 and IL-5 were significantly increased in the model group compared with the control group, and IFN-γ was decreased compared with the control group, indicating that the AR model was deviated from Th2. However, the high and moderate doses of AEO can inhibit the migration of Th2 to different degrees, and promote the formation of a new balance of Thl. Cet group also inhibited the chemotaxis of Th cells to some extent, but had no obvious effect on IL-5, suggesting that the effect of Cet on AR retardation phase may be less than that of AEO.

Thl7 cells have also been reported to play an important role in the development of Th2-mediated allergic diseases, but current studies suggest that IL-17 may promote neutrophil aggregation by inducing IL-8 or mast cell activation [30]. In this study, the level of IL-17 in AR model group was significantly higher than that in control group, and both AEO and Cet could reduce the level of serum IL-17 in some extent.

The present study clarified the anti-allergic effect of AEO and preliminarily discussed its mechanism. AEO can control the development of AR by regulating T-cell immune response, down-regulating IgE, inhibiting eosinophil infiltration and so on, which reflects the multicomponent, multitarget, and multilink characteristics of traditional Chinese medicine. However, the mechanism and important pathways of AEO need further in-depth study.

## Data Availability

Data will be made available from the corresponding author to all interested researchers upon reasonable request.

## Abbreviations

AEO: *Asarum sieboldii* Miq. essential oil
AR: allergic rhinitis
Cet: cetirizine
H&E: Hematoxylin–Eosin
IFN: Interferon
IgE: immunoglobulin E
IL: Interleukin
OVA: ovalbumin
XX: xixin
