# Expression of Concern: Protective effect of Asarum sieboldii essential oil on ovalbumin induced allergic rhinitis in rat

**DOI:** 10.1042/BSR-20191370_EOC

**Published:** 2020-06-22

**Keywords:** Nasal mucosa, sneezing, nasal scratching, histamine, cytokines

The Editorial Office has been made aware of potential issues surrounding the scientific validity of this paper, hence has issued an expression of concern to notify readers whilst the Editorial Office investigates.

